# Increasing Co-occurrence of Additional Autoimmune Disorders at Diabetes Type 1 Onset Among Children and Adolescents Diagnosed in Years 2010–2018—Single-Center Study

**DOI:** 10.3389/fendo.2020.00476

**Published:** 2020-08-06

**Authors:** Barbara Głowińska-Olszewska, Maciej Szabłowski, Patrycja Panas, Karolina Żoła̧dek, Milena Jamiołkowska-Sztabkowska, Anna Justyna Milewska, Anna Kadłubiska, Agnieszka Polkowska, Włodzimierz Łuczyński, Artur Bossowski

**Affiliations:** ^1^Department of Pediatrics, Endocrinology, Diabetology With Cardiology Division, Medical University of Bialystok, Białystok, Poland; ^2^Department of Pediatrics, Rheumatology, Immunology and Metabolic Bone Diseases, Medical University of Bialystok, Białystok, Poland; ^3^Department of Statistics and Medical Informatics, Medical University of Bialystok, Białystok, Poland; ^4^Department of Medical Simulations, Medical University of Bialystok, Białystok, Poland

**Keywords:** diabetes type 1, autoimmune thyroid diseases, celiac disease, children, epidemiology

## Abstract

**Objectives:** The prevalence of type 1 diabetes mellitus (T1D) in children is growing, but its relation to other autoimmune disorders that coexist since the onset of diabetes is not recognized. The objective of this study was to assess the incidence of T1D and the prevalence of autoimmune illnesses additionally coexisting since the diabetes mellitus onset in children during a period of 9 years' observation.

**Methods:** In this retrospective study, the incidence rate (IR) of the T1D was calculated as the total number of all cases that were newly diagnosed per 100,000 population people between 0 and 18 years of age. The selected age groups (0–4, 5–9, 10–14, and 15–18 years) were examined, respectively. The studied group included 493 children (264 [53.55%] boys) between 0 and 18 years old newly diagnosed with T1D in one of the Polish centers in the years 2010–2018. Other autoimmune illnesses diagnoses were obtained from medical records taken from the first hospital treatment, when T1D was recognized.

**Results:** The annual standardized IR of T1D increased from 19.2/100,000 in year 2010 to 31.7/100,000 in 2018 (1.7-fold over 9 years' observation), with an increase in the incidence rate ratio (IRR) by 4% per year. The highest growth in IR was recorded in 5- to 9-year-olds (from 19.61 in 2010 to 43.45 in 2018). In 61 (12.4%) of the studied group, at least one additional autoimmune disease was diagnosed. The prevalence doubled from 10.4% in the year 2010 to 20.8% in the year 2018. Autoimmune thyroid illnesses were found in 37 children (7.5%); their incidence increased from 6.3% to almost 2-fold, 12.5%, in 2018. In 26 children (5.3%), celiac disease was recognized; the prevalence increased from 4.2 to 9.8% in the study period. The prevalence of additional autoimmune thyroid disease was higher in glutamic acid decarboxylase–positive antibodies (χ^2^ = 3.4, *p* = 0.04) patients, the oldest age group (15–18 years) (χ^2^ =7.1, *p* = 0.06), and in girls (χ^2^ =7.1, *p* = 0.007).

**Conclusions:**The standardized IR of T1D in children increased 1.7-fold over the 9-year observation period, and IRR increased 4% per year. Additional autoimmunity represents a significant comorbidity in patients with new-onset T1D. The number of children diagnosed with additional autoimmune diseases that accompany T1D is rapidly growing in all age groups throughout recent years.

## Introduction

The prevalence of type 1 diabetes mellitus (T1D) has received much attention lately, and the rapid increase in the number of patients should not be disregarded. According to the ninth edition of *IDF Diabetes Atlas* 2019 (1), it is estimated that 1,110,100 young people younger than 20 years have T1D worldwide. Type 1 diabetes incidence in children increases by 2–5% annually worldwide, according to large epidemiologic studies (2), and it depends on the geographic region; for example, in Asian countries, the incidence rates (IRs) are usually very low (3), whereas the rates in some European countries, for example, Finland, are indisputably high (4). In European cases, the incidence among girls is currently the highest in the age group between 5 and 9 years, whereas that of boys is highest in the 10- to 14-years age group (5). In years 2010–2014 in Poland, incidence rate ratio (IRR) increased 1.5-fold (by 12.73% annually) in children and adolescents aged 0–17 years (6).

It has been reported recently, among them in meta-analysis, that frequency of autoimmune diseases (AD) in general, has increased significantly over the last 30 years, with thorough research of risk factors and environmental impacts on susceptibility to AD (7, 8). The global prevalence of AD in pediatric age is ~5%, and the most frequent autoimmunities are represented by autoimmune thyroid diseases (AITDs) (9). The incidence of autoimmune hypothyroidism in children is rated at 1–2% with a dominance of female 4:1 (10). Even subclinical course of the disease is known to result in many adverse effects such as increased risk of congestive cardiac failure and coronary heart disease events. What is more, subclinical hypothyroidism is likely to cause cognitive impairment and non-specific symptoms, for example, fatigue and mood changes (11). Hyperthyroidism constitutes 15% of children's thyroid disorders, and most of the cases can be attributed to hyperthyroidism of autoimmune origin, Graves disease (GD) (10).

It is now recognized that the prevalence of additional autoimmunity is increasingly encountered in clinical practice of pediatric diabetologists. Additional ADs (AADs) frequently occur in the same individual over the course of T1D, suggesting strong shared genetic susceptibility and pathological mechanisms. Patients with T1D demonstrate an increased exposure to other autoimmune disorders, for example, AITD (Hashimoto thyroiditis and GD, 15–30%), Addison disease (0.5%), autoimmune gastritis (5–10%), celiac disease (CD; 4–9%), and vitiligo (2–10%) (12, 13). Revealed high prevalence of associated autoimmune conditions generated the need for early screening of these diseases (14).

There is increasing knowledge about the prevalence of AAD in the course of long-lasting T1D in children with T1D (15, 16). Notably, the association of T1D with other autoimmune illnesses that coexist from the onset of diabetes and the actual trend of this connection over the years are not fully recognized. We aimed to estimate the prevalence of patients diagnosed with the most common autoimmune disorders: AITD and CD at the diagnosis of T1D and the changes in prevalence of these diseases during 9 years (2010–2018) observation. Second, we intended to search for possible clinical factors connected with multiple autoimmunities since T1D onset. We hypothesized that together with increasing prevalence of diabetes type 1 in children over the years, the number of patients diagnosed with AAD will also increase.

## Patients and Methods

The study was planned as a retrospective, hospital records–based study including 493 children and adolescents (264 boys and 229 girls, aged 0–18 years), who were diagnosed for T1D between 2010 and 2018 at the Department of Pediatrics, Endocrinology, Diabetology with Cardiology Division, Medical University of Białystok, Poland. This is the only center for diabetic children in the Podlasie Voivodeship Region (northeast part of Poland), where all young patients with newly diagnosed T1D from the region are treated. The IR of the T1D was calculated as the total number of all cases that were newly diagnosed per 100,000 people between 0 and 18 years of age. General population figure was obtained from the Central Statistical Office of Poland (Polish: Glowny Urzad Statystyczny) (17). The selected age groups (0–4, 5–9, 10–14, and 15–18 years) were examined separately. Laboratory and anthropometric data were obtained from electronic medical records, body mass index (BMI), and standard-deviation-score (SDS) for BMI was calculated using age- and sex-specific BMI growth charts according to local Polish OLAF study (18).

The diagnosis of T1D was made on the basis of criteria for diabetes type 1 recognition according to the International Society for Pediatric and Adolescent Diabetes guidelines: history of polyuria, polydipsia, and weight loss with an elevated random plasma glucose of ≥11.1 mmol/L or fasting plasma glucose of ≥7 mmol/L (19). Diabetic autoimmunity was confirmed on the basis of at least one positive titer of autoantibodies to islet cells (ICAs), glutamic acid decarboxylase (GADA), and also the protein tyrosine phosphatase (IA2). Analysis was performed in the same laboratory for all the study period. The diagnosis date referred to the date of the very first injection of insulin, according to EURODIAB criteria (20), and our previous publications. The age of the patients was calculated in completed years on the day of T1DM diagnosis (21, 22).

Serologically screening tests for thyroid autoimmunity included anti-thyroid peroxidase antibodies (aTPO) and antithyroglobulin (aTg), and, in cases of hyperthyroidism suspicion, anti-thyroid stimulating hormone (TSH) antibodies (TR-Ab). Confirmation of thyroid autoimmunity was based on the elevated titer of aTPO, aTg, or TR-Ab. Both hypothyroid and hyperthyroid autoimmunity was taken into consideration. Children were screened for the thyroid diseases using TSH and free thyroxine (fT4) levels at the moment of diagnosing. Furthermore, in each case of newly diagnosed T1D, according to our local recommendations, thyroid ultrasound has been performed since 2016. The clinical entities found in AITDs are diverse and vary depending on whether it is in a state of autoimmune hypothyroidism (HT, Hashimoto disease) or hyperthyroidism (GD). Patients suffering from HT represented clinical and biochemical characteristics of hypothyroidism and demonstrated an elevated TSH with presence of elevated anti-TPO and/or aTg autoantibodies. Graves disease was diagnosed in children with large goiter, hyperthyroidism in laboratory tests and positive thyrotropin receptor (TR-Ab) antibodies, anti-TPO antibodies, and aTg antibodies. Subclinical hypothyroidism, without confirmed autoimmunity, was not taken into consideration into further analyses. The diagnosis of AITDs was set on the basis of clinical, laboratory, and ultrasound investigations and was always set with experienced pediatric endocrinologist, and pharmacological treatment was introduced if needed.

To diagnose celiac autoimmunity, anti-tissue transglutaminase (anti-tTGA) antibodies were performed. With titer of ≥10 IU/mL or with questionable results, anti-endomysial (EMA) and anti-reticulin (ARA) antibodies were also implemented. In this case, small intestine–associated autoimmunity was recognized. The CD was diagnosed on the basis of the revised criteria of CD diagnosis according to the European Society Pediatric Gastroenterology, Hepatology, and Nutrition criteria (21, 23) and always consulted with experienced gastroenterologist. Patients positively serologically tested for tTGA, EMA, and ARA had a gastroscopy performed if needed for diagnosis. Children recognized with the disease started gluten-free diet since diabetes onset. We did not test our patients for Addison disease because it is out of obligatory screening in Poland. Yet, none of the children represented clinical symptoms of this or any other ADs that may accompany T1D (gastritis, vitiligo, etc.).

### Laboratory Analyses

The analyses were performed with routine laboratory methods in hospital laboratory on an ongoing basis. Fasting blood sample for analysis was collected in the morning. Serum levels of TSH, fT4, and triiodothyronine (fT3) were assessed with the use of electrochemiluminescence “ECLIA” with Cobas E411 analyzer (Roche Diagnostics, Warsaw, Poland). Values within the norm presented the range between 0.28 and 4.3 μIU/L for TSH, between 1.1 and 1.7 ng/dL for fT4, and between 2.6 and 5.4 ng/dL for fT3. Anti-TPO, aTg, and TR-Ab antibodies were analyzed in all samples with the use of ECLIA with Modular Analytics E170 analyzer (Roche Diagnostics). The positive values for antithyroid antibodies were >1.75 U/L for TR-Ab, >34 IU/mL for anti-TPO-Abs, and >115 IU/mL for aTg-Abs. Tests for beta cells and thyroid and celiac antibodies were performed after recovery from ketoacidosis and the initiation of subcutaneous insulin therapy, usually between the fifth and the seventh day of the child's stay in the ward (in our hospital, hospitalization usually lasts 8–10 and is related to patient's education).

The study was performed in accordance with the Guidelines of Good Clinical Practice. The protocol was approved by the Medical University in Białystok, Poland Bioethical Committee. Parents/legal guardians and their children both provided their written informed consent. The study adhered to ethical standards including ethics committee approval and consent procedure. Also, standard biosecurity and institutional safety procedures were adhered to.

### Statistical Analysis

The IR of the T1D was calculated as the total number of all cases that were newly diagnosed per 100,000 population people at 0- to 18-year age per year. A multifactorial Poisson regression model was made to assess the IRR, depending on age and year. For the needs of the model for age, dummy variable was created, in which the 0- to 4-year age group is the base (reference) variable. Model fit was assessed using the Pearson goodness-of-fit test. One-way linear regression models were used in the time trend analysis. For IRR by age group and year, exact Poisson 95% confidence intervals (CIs) were calculated. The IRs were directly standardized by age according to the population of Poland 2010. The prevalence of T1D and additional AID was evaluated using χ^2^ or Fisher exact test when appropriate. A multiple logistic regression analysis was used to recognize independent factors and their influence on additional autoimmunity prevalence. The odds ratios with 95% CIs were calculated. *p*-value lower than 0.05 was determined as statistically significant. The results are presented as mean ± SD, or numbers (*n*), and percentages (%). Statistically significant results were found at *p* < 0.05. The data were calculated using Stata/IC 12.1 package from StataCorp LP, College Station, TX, USA and STATISTICA 13.0 software, StatSoft Polska Sp. z o.o. Kraków, Poland.

## Results

**Figure 1:**
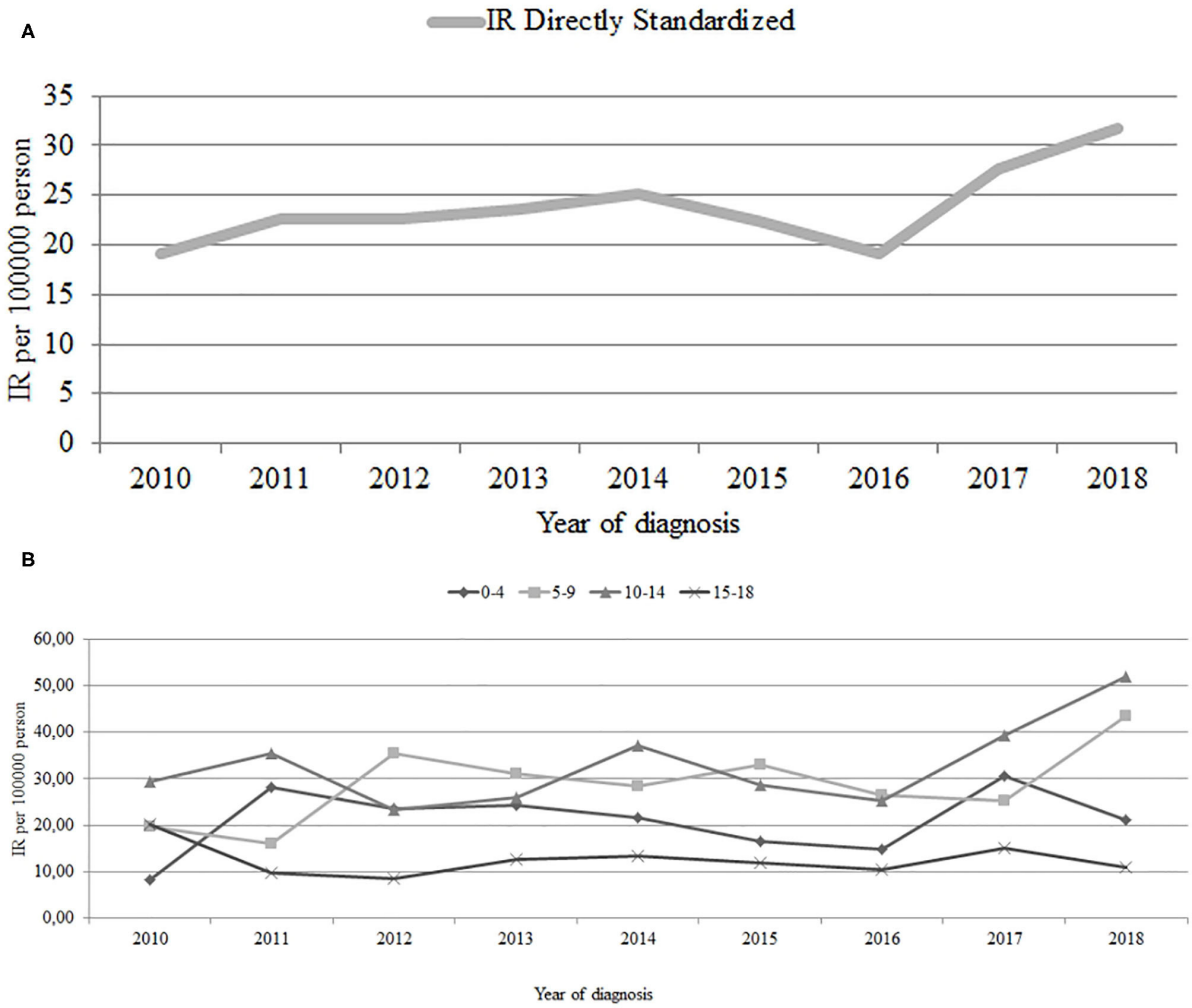
**(A)** T1D IR Directly Standardized in years 2010–2018 in all age groups (0–18 y.o.) A *incidence rate directly standardized* is the number of new cases occurring in a specified population during a year, expressed as the number of cases per 100,000 population of Podlaskie Voivedeship aged 0–18 yrs, standardized to the age-matched population of Poland from 2010. Slope = 0.948, *P* = 0.057. **(B)** T1D incidence rate (IR) in years 2010–2018 in certain age groups. A *crude incidence rate* is the number of new cases occuring, in a specified population during a year, expressed as the number of cases per 100,000 population of Podlaskie. Voivodeship in these age groups. 0–4 y.o. slope = 0.561, *p* = 0.566. 5–9 y.o slope = 1.78, *p* = 0.095. 10–14 y.o slope = 1.82, *p* = 0.127. 15–18 y.o slope = 1.78, *p* = 0.521.

We analyzed the data from total 493 pediatric patients (264 male and 229 female) aged 0 to 18 years [median (interquartile range) of age at diagnosis 9.50 (5.50–12.50) years], diagnosed successively between 2010 and 2018. During the study period, 23 children were diagnosed with other types of diabetes (11 with maturity-onset diabetes of the young 2, six with type 2, and six were insulin dependent, but autoantibody negative—not included into the present analysis as T1D). Two of our patients were diagnosed with AITD, and one, CD before the diagnosis of T1D. The general characteristics of the studied group are presented in Table 1. Mean IR of T1D among children and adolescent patients during the study period was 23.78/100,000 age-matched population. We found that incidence of diabetes mellitus type 1 in Podlaskie Voivodeship keeps rising. During the time of the study, the age-standardized annual incidence increased from 19.2/100,000 in 2010 up to 31.7/100,000 in 2018 over 9 years, in total 1.7-fold in the observed period. Annual fluctuations, with the lowest IR in 2016, were noticed. Time trend analysis revealed increase in incidence ratio (slope 1.06, *p* = 0.039), although statistically insignificant when standardized for age (*p* = 0.057) (Figure 1A). The increase in the IRR was 4% per year.

**Table 1:** General characteristics of the study group.

**Parameters at diagnosis**	**No. of valid results**	**% of valid results**	**Median [interquartile range]**	**Minimal value**	**Maximal value**
Age at diagnosis (years)	493	100.00	9.50 [5.50–12.50]	0.50	18.00
0–4			*n* = 109		
5–9			*n* = 151		
10–14			*n* = 172		
15–18			*n* = 61		
HbA_1c_ (%)	369	75	10.70 [9.46–12.42]	6.00	21.0
pH	487	99	7.37 [7.28–7.41]	6.93	7.64
HCO_3_	220	45	16.60 [8.50–20.95]	2.60	43.4
C-peptide (ng/mL)	295	60	0.47 [0.28–0.79]	0.00	1.1
GADA (U/mL) [86.82%—positive]	433	88	63.20 [8.67–280.03]	0.27	5,775
IA2A (U/mL) [86%—positive]	434	88	188.05 [8.16–777.51]	0.00	3,626
ICA (JDF U) [64.91%—positive]	431	87	20.00 [10.00–40.00]	0.00	640
TPO (IU/mL)	474	96	5.00 [5.00–6.90]	5.00	600.00
ATG (IU/mL)	474	96	10.00 [10.00–10.00]	3.15	4,000.00
TTGA (UI/mL)	461	93	10.00 [10.00–10.00]	0.4	1,700
BMI	470	95	15.7 [14.2–18.1]	8.9	35.01
SDS-BMI	429	87	−0.43 [−1.08 to 0.29]	−3.40	5.07
Gender (%), *n*	Boys: 53.55% *n* = 264		Girls: 46.45% *n* = 229		

We divided the studied population into certain age groups and assessed the IR one by one. We discovered the growth in three of the age groups (from 8.22 to 21.08 in group 0–4 years old; from 19.61 to 43.45 in group 5–9 years old; from 29.26 to 52.01 in group 10–14 years old) and decrease in the oldest (15–18 years) age group of patients, from 20.16 to 10.95. The overall upward trend in incidence of T1DM seems to come from the increase in groups 5–9 years old and 10–14 years old, in which the rise in IR was the greatest. The lowest mean IR was found in group 15–18 years old (12.52/100,000), and the highest IR was noticed in group 10–14 years old (32.89/100,000). In other age groups, it was, respectively, 20.95 in group 0–4 years old and 28.78 in group 5–9 years old. In the time trend analysis, we did not prove significant differences when selected age groups were studied (Figure 1B). The Poisson regression model adjusted for the year of diagnosis and age group demonstrated the incidence risk ratio increasing 1.37 in children aged 5–9 years, 1.57 for children aged 10–14 years, and 0.61 for children aged 15–18 years. The year of diagnosis increased the IRR 1.04 times (4%) (Table 2). The differences in the standardized incidence ratios of diagnosed T1D cases between male and female were not statistically different and are presented in Table 3. In Supplementary Table 1, we included the data of incidence ratios with 95% CI in separated age groups in every studied year.

**Table 2:** Model of Poisson regression for IRR based on a group of 0–4 years old.

**Age group (years)**	**IRR (95% CI)**	***P***
0–4	Base	
5–9	1.37 (1.06–1.75)	0.013
10–14	1.57 (1.23–1.99)	0.000
15–18	0.61 (0.44–0.83)	0.002
Year	1.04 (1.00–1.07)	0.021

*Pearson goodness of fit = 33.0039*.

**Table 3:** Age-standardized incidence rates (per 100,000 person-years) of type 1 diabetes according to gender.

	**IR (95% CI) age standardized**		
**Year**	**Girls**	**Boys**	**Total**
2010	17.5 (9.8–24.4)	21.2 (13.2–29.2)	19.2 (13.7–24.6)
2011	24.2 (15.2–22.2)	21.3 (13.1–29.4)	22.7 (16.6–28.8)
2012	20.9 (12.5–29.3)	24.2 (15.4–33)	22.6 (16.5–28.7)
2013	21.3 (12.6–29.6)	25.5 (16.4–24.6)	23.5 (17.2–29.7)
2014	23.3 (14.2–32.2)	26.9 (17.4–36.4)	25.1 (15.6–31.7)
2015	19.9 (11.5–28.3)	24.7 (15.7–33.8)	22.4 (16.2–28.3)
2016	21.6 (12.9–30.2)	16.7 (9.2–24.3)	19.1 (13.20–24.8)
2017	29.6 (19.2–39.9)	25.9 (16.5–35.4)	27.7 (20.7–34.7)
2018	25.2 (15.9–34.6)	37.9 (26.7–49.2)	31.7 (24.4–39.1)

**Figure 2:**
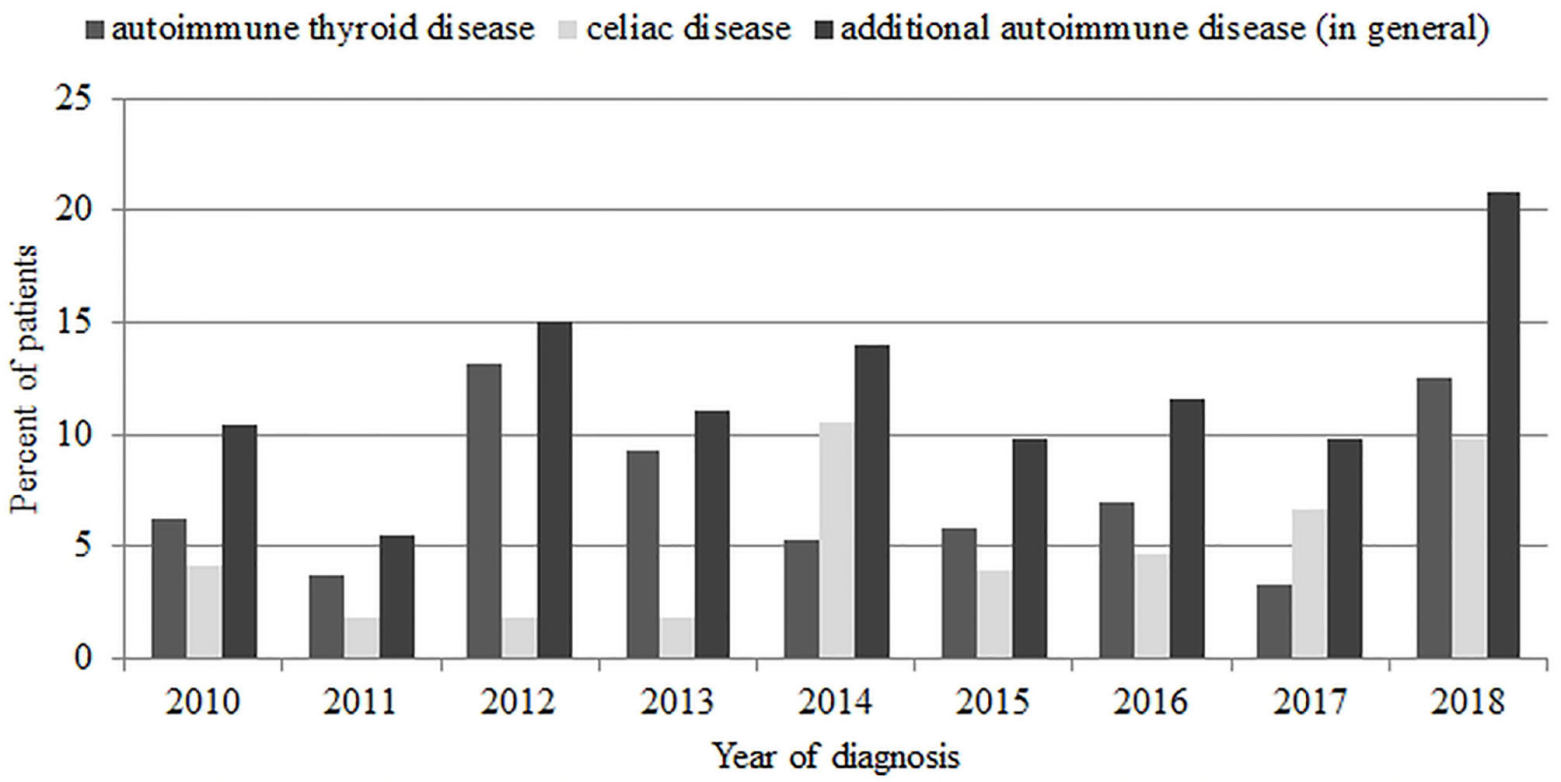
Prevalence of additional autoimmune diseases (autoimmune thyroid disease and celiac disease) in new-onset type 1 diabetes mellitus patients.

Next, we assessed the prevalence of additional autoimmunity and AD in all new-onset cases of T1D in subsequent years of analysis. In total, thyroid autoimmunity was recognized in 66 patients (13.39%), and AITD was recognized in 37 patients (7.51%) during the study period. Celiac autoimmunity was recognized in 38 patients (7.7%), whereas CD was found in 26 patients (5.3%). We found that general specific autoimmunity (other than diabetic antibodies) at T1D diagnosis remained stable during the study period (from 25% of patients in 2010 up to 23.6% in 2018; χ^2^ = 1.2 *p* = 0.26). As for AADs (celiac and AITD), their prevalence fluctuated during the study: it started from 10.4% in 2010, next increased in years 2012–2014, and then fell in years 2015–2017 to eventually finish at 20.83% in 2018. In the end, this gives a 2-fold increase in the percentage of patients with AADs during the 9-year study period (Figure 2, Table 4).

**Table 4:** Other organ autoimmunity and autoimmune diseases among DMT1 patients aged 0–18 years diagnosed in years 2010–2018.

**Annual distribution**												
**Year**	**Total**	**2010**	**2011**	**2012**	**2013**	**2014**	**2015**	**2016**	**2017**	**2018**	***χ***^**2**^ **and** ***p*****-value total**	***χ***^**2**^ **and** ***p*****-value 2010 vs. 2018**
T1D, *n*	493	48	54	50	54	57	51	43	61	72		
TAI, *n* (%)	66 (13.5)	8 (16.6)	10 (18.5)	9 (18.0)	8 (14.8)	6 (10.5)	6 (11.8)	4 (9.3)	5 (8.2)	10 (13.8)	χ^2^ = 5.2 *p* = 0.7	χ^2^ = 2.3 *p* = 0.12 *Y* = 1.6 *p* = 0.19 Fi *p* = 0.09
TTGA, *n* (%)	38 (7.8)	5 (10.4)	7 (13.7)	1 (1.9)	3 (5.6)	6 (10.5)	3 (5.9)	2 (4.6)	4 (6.5)	7 (9.8)	χ^2^ = 7.8 *p* = 0.45	χ^2^ = 0.4 *p* = 0.50 *Y* = 0.09 *p* = 0.70 Fi *p* = 0.30
AI, *n* (%)	99 (20.1)	12 (25)	15 (27.7)	10 (18.8)	10 (18.5)	11 (19.3)	9 (17.6)	6 (13.9)	9 (14.7)	17 (23.6)	χ^2^ = 5.7 *p* = 0.68	χ^2^ = 1.2 *p* = 0.26 *Y* = 0.7 *p* = 0.37 Fi *p* = 0.18
AITD, *n* (%)	37 (7.5)	3 (6.25)	2 (3.7)	7 (13.2)	5 (9.3)	3 (5.3)	3 (5.9)	3 (7)	2 (3.3)	9 (12.5)	χ^2^ = 8.7 *p* = 0.36	χ^2^ = 1.2 *p* = 0.20 *Y* = 0.6 *p* = 0.40 Fi *p* = 0.20
CD, *n* (%)	26 (5.3)	2 (4.2)	1 (1.8)	1 (1.9)	1 (1.8)	6 (10.5)	2 (3.9)	2 (4.6)	4 (6.7)	7 (9.8)	χ^2^ = 10.4 *p* = 0.2	χ^2^ = 1.3 *p* = 0.20 *Y* = 0.6 *p* = 0.42 Fi *p* = 0.21
AAD, *n* (%)	61 (12.4)	5 (10.4)	3 (5.6)	8 (15.1)	6 (11.1)	8 (14.0)	5 (9.8)	5 (11.6)	6 (9.8)	15 (20.8)	χ^2^ = 8.5 *p* = 0.38	χ^2^ = 2.7 *p* = 0.09 *Y* = 1.9 *p* =1.50 Fi *p* = 0.07

*n, no. of patients; X^2^ - Chi-squared; Y, Yates correction; Fi, Fisher exact test; T1D, type 1 diabetes mellitus; TAI, thyroid-positive autoimmunity; TTGA, anti-tissue transglutaminase antibodies positive; AI, autoimmunity (thyroid + celiac); AITD, autoimmune thyroid disease; CD, celiac disease; AAD, additional autoimmune disease (total)*.

**Figure 3:**
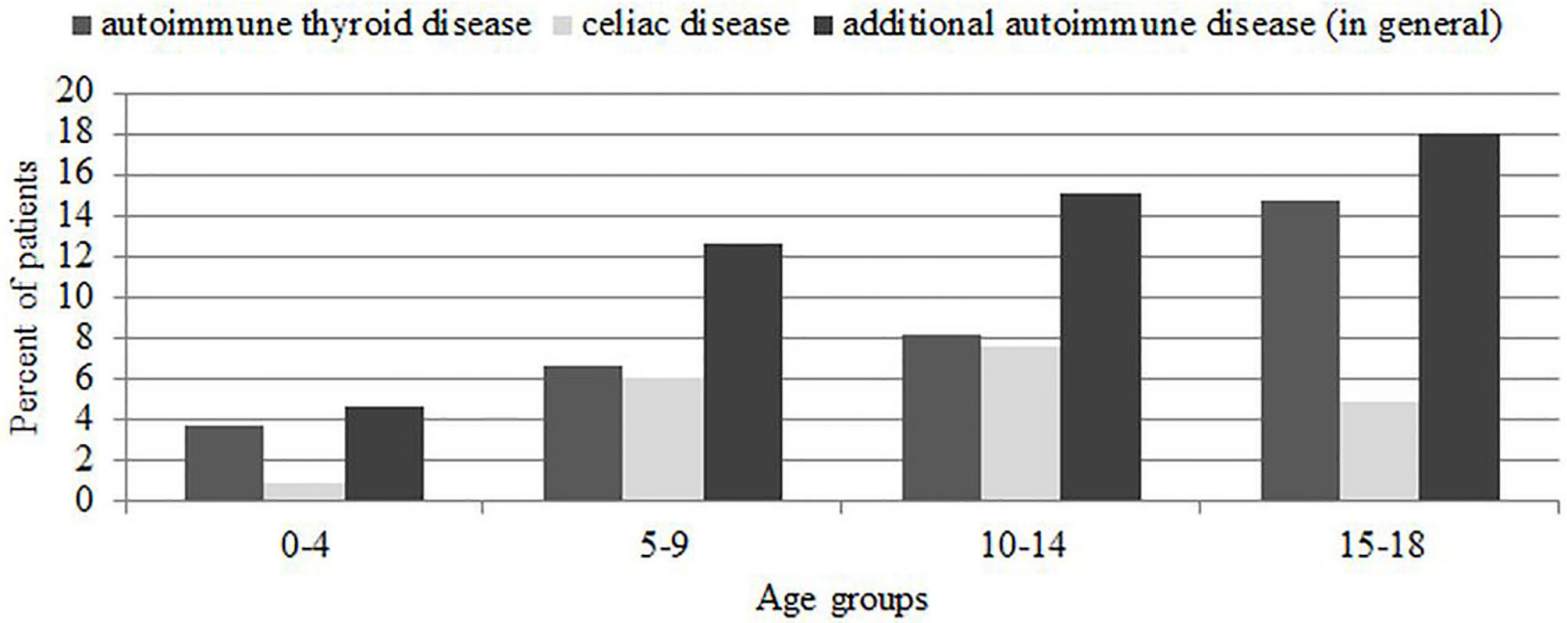
Prevalence of autoimmune thyroid disease and celiac disease in new-onset T1D. Comparison between age groups.

**Figure 4:**
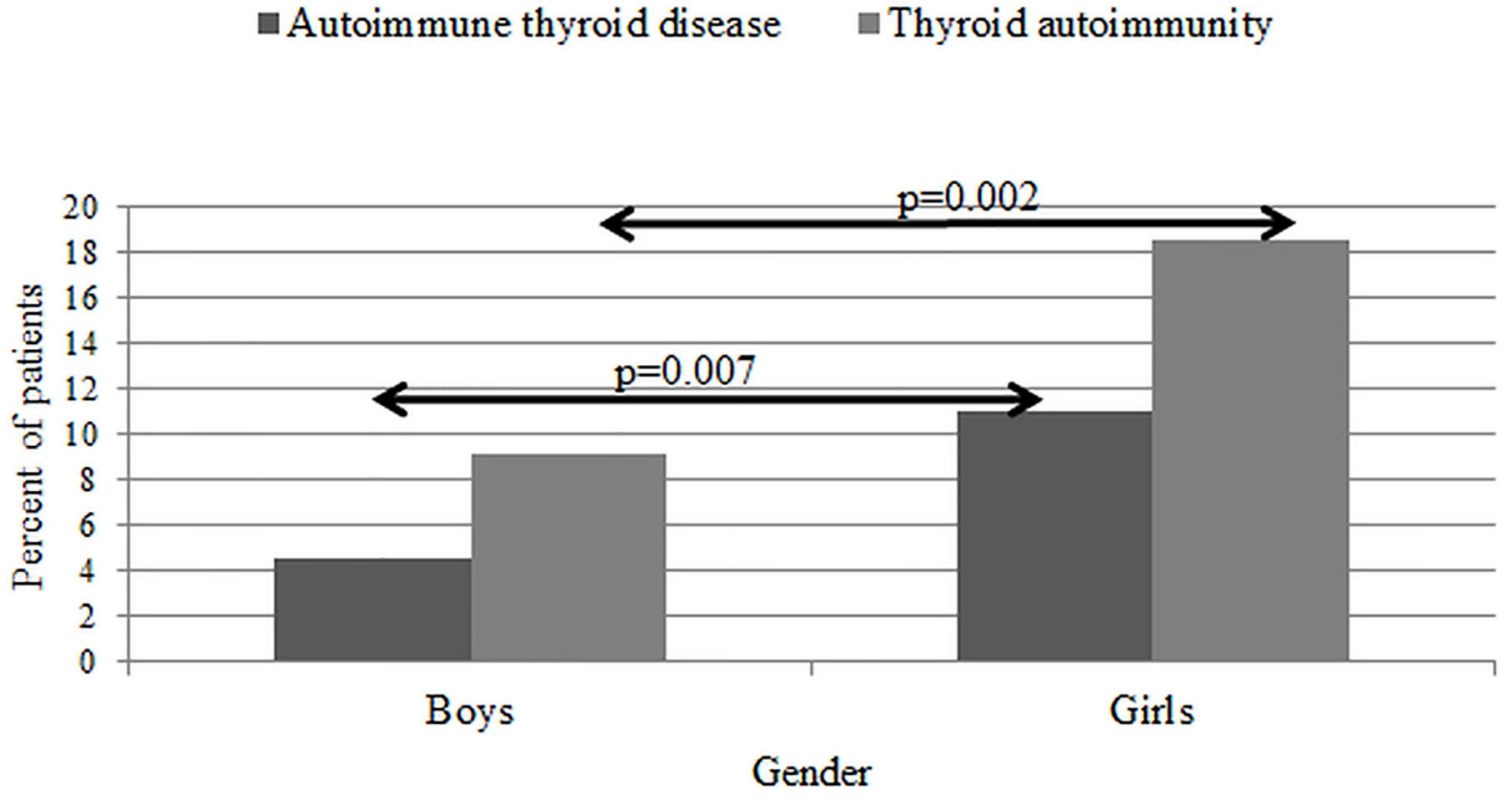
Prevalence of patients with thyroid autoimmunity and autoimmune thyroid disease at diabetes type 1 diagnosis.

In further analysis, considering the frequency of autoimmunity in T1D age groups, we did not show statistically significant differences (χ^2^ = 1.7, *p* = 0.63). As for AITD, it was similar; we found higher prevalence of these diseases in the oldest age group, although it was not statistically significant (χ^2^ = 7.1, *p* = 0.06) (Figure 3). The detailed comparison of the differences in mean age between additional autoimmunity “positive” and “negative” patients is presented in Table 5. We found that patients with additional autoimmmune problem, especially thyroid disease, were significantly older. Thyroid autoimmunity varied significantly between male (9.13%) and female (18.5%) (χ^2^ = 9.2, *p* = 0.002), as well as thyroid AD (male 4.55%, female 10.92%, χ^2^ = 7.2, *p* = 0.007) (Figure 4). Such differences were not found for CD.

**Table 5:** Mean age at diagnosis of T1D in the groups of “positive” and “negative” patients in terms of total autoimmunity, AITD, thyroid autoimmunity, celiac disease, TTGA autoimmunity, and additional autoimmune disease.

	**Additional autoimmunity “positive” patients**	**Additional autoimmunity “negative” patients**	***p*-value**
	**Mean age**		
Autoimmunity	10.13 ± 4.42	8.80 ± 4.58	0.009
AITD	11.14 ± 4.33	8.91 ± 4.56	0.004
Thyroid autoimmunity	10.14 ± 4.68	8.92 ± 4.55	0.040
Celiac disease	10.69 ± 3.52	8.98 ± 4.62	0.064
TTGA autoimmunity	10.03 ± 4.29	9.02 ± 4.59	0.203
Additional autoimmune disease	10.70 ± 3.96	8.85 ± 4.62	0.004

**Figure 5:**
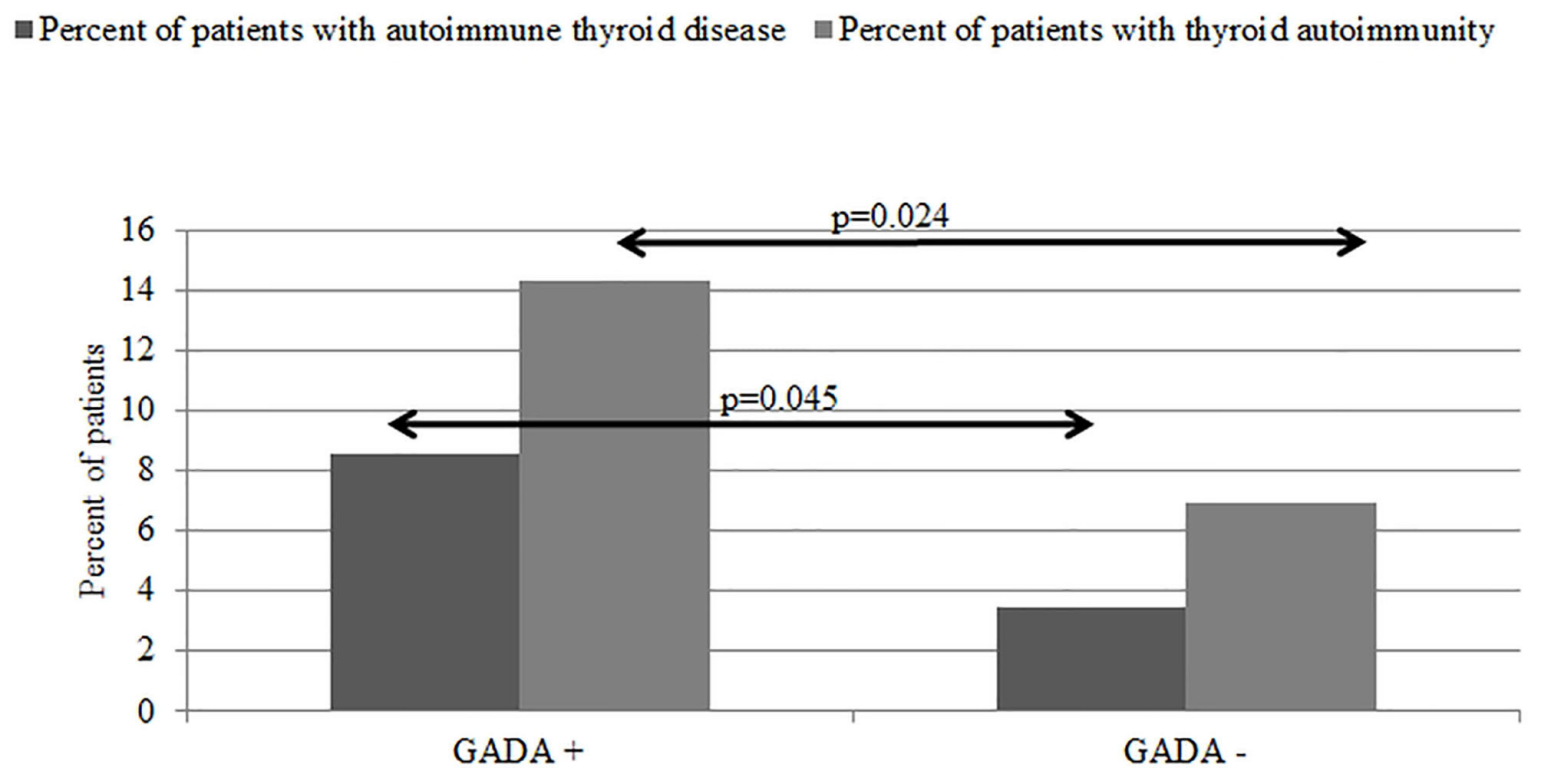
Prevalence of autoimmune thyroid disease and thyroid autoimmunity in patient with positive or negative GADA autoantibodies.

Finally, we analyzed the prevalence of AAD according to the presence of GADA antibodies. We discovered that children with positive anti-GAD antibodies were much more likely to develop thyroid autoimmunity at diagnosis of T1D than patients with negative anti-GAD, and this difference was observed as statistically significant (*p* = 0.024). We also obtained a significant difference in frequency of AITD between both groups (*p* = 0.045). As presented in Figure 5, in GADA(+) patients, 8.54% had AITD, and 14.33% had thyroid autoimmunity at diagnosis of T1D, whereas in GADA(–) patients, it was 3.42 and 6.9%, respectively, *p* < 0.05 (Figure 5). We have also assessed the possible relationship between the age of patients and the titers of GADA, ICAs, IA2, and antithyroid antibodies without showing a statistically significant difference (Student *t*-test; data not shown).

Furthermore, we performed a multiple logistic regression analysis, which showed that female gender (*p* = 0.01), year of onset (*p* = 0.01), and GADA positive (*p* = 0.04) influenced the presence of additional thyroid autoimmunity at diabetes type 1 onset. In this analysis, we did not confirm the independent influence for the age at onset. As for AITD, the independent influencing factors were female gender (*p* = 0.03), GADA-positive antibodies (*p* = 0.01), and age at onset (*p* = 0.01) (Table 6).

**Table 6:** Independent factors influencing additional thyroid autoimmunity and thyroid disease at diabetes type 1 onset in a multiple logistic regression analysis.

	**OR**	**95% CI**	***p*-value**
**Additional thyroid autoimmunity**			
Gender	1.9	(1.1–3.4)	0.01
Year of onset	0.9	(0.7–1.0)	0.01
GADA antibodies	1.0	(1.0–1.0)	0.04
Age at onset	1.2	(0.9–1.6)	0.09
**Autoimmune thyroid disease**			
Gender	1.9	(0.9–4.0)	0.03
Year of onset	1.0	(0.9–1.1)	0.4
GADA antibodies	2.1	(0.7–6.1)	0.01
Age at onset	1.6	(1.1–2.3)	0.01

*CI, confidence interval; OR, odds ratio*.

Autoimmune hyperthyroidism was found in three of our patients. Their cases were included into the AITD group. Four of the children had developed both CD and AITD; they were treated separately in the analyses. None of our patients represented clinical symptoms of any other autoimmune disorder.

## Discussion

In our study, we presented high, in our opinion, overall prevalence of AADs (thyroid and celiac) in children and adolescents who newly received the diagnosis of T1D. Our results showed increasing T1D IRs over the observed years, accompanied with growing prevalence of other coexisting ADs, with the 2-fold increase in AITD. To the best of our knowledge, this work represents the first report considering the epidemiological trends and distribution by demographic data (gender and age) of the most common AAD—AITD and CD—among newly diagnosed T1D pediatric patients. Therefore, we believe that our study may provide new insight into the relation of T1D and other autoimmunities in pediatric population. Moreover, we reported significant association between specific diabetic autoimmunity (GADA) and female preponderance with increased prevalence of thyroid autoimmunity and AITD. Last but not least, we observed a trend for increase of AAD at T1D onset with the older age at diagnosis.

In our present results, we observed increasing T1D incidence over the observed 2010–2018 years; the overall IRR amounted to 4% a year (1.7-fold), and we noticed the highest IR in children aged 10–14 years. According to the latest data, incidence of T1D is increasing irrespectively of the genetics characteristic for the region and geographical location. Numerous research tried to find demographic trends concerning age and gender (6, 24). The recent literature emphasizes the trend of increased prevalence of T1D among all age groups of people (25–27). Observed worldwide IR of T1D incidence in children is increasing by 2–5% annually. Nevertheless, IRs vary among regions of the world. In Asia, the incidence of T1D is very low: China (Shanghai), 3.1 per 100,000 (28). Some research was made, looking for a possible link between these differences in IR and HLA specific to Caucasians and Asian populations (29, 30). In Europe, newly diagnosed cases of T1D are increasing by 3.4% per year (25). According to the large nationwide studies, in Poland, incidence of T1D is also increasing year after year (6). In the previous study considering our region, the lowest IR was observed in the youngest group of children aged (0–4 years), whereas the highest IR was identified in older children (10–14 years) (21). American SEARCH for Diabetes in Youth, a multicenter study of American population, also confirmed this phenomenon (an incidence of 33.9/100,000 between 10 and 14 years old, regardless of ethnic group and region of residence). The researchers link this to an increase in insulin resistance during puberty and pathogenic impact of infections (31).

One of the main findings of our study is the general increase in prevalence of AAD at T1D onset. During the study period, the percentage of patients diagnosed with CD and AITD doubled, with the greatest rise between 2017 and 2018. Numerous studies reported that incidence and prevalence of autoimmunity and AID in diabetic patients increase with age and are more common in females (32, 33), what we also confirmed in our research. Although statistically significant differences were noted only regarding AITD and thyroid autoimmunity, for autoimmunity connected to CD, we observed only percentage differences.

The frequency of AITD in a combined population of Europe is calculated as 3% for hypothyroidism and 0.75% for hyperthyroidism (34). Autoimmune thyroid disease affects as much as up to 3% of the pediatric population, so it represents the example of the most common ADs (9). In T1D, patients' reported weighted mean prevalence of hypothyroidism was 9.8%, whereas in hyperthyroidism it was 1.3% according to one of the recent meta-analyses (35), so it is significantly increased compared to general population. What is more, we could speculate that if prevalence of AITD increases during the disease (36), and we noticed incidence >20% at diagnosis in 2018, the prevalence of AITD in children diagnosed in 2018 may in the further course of the disease exceed the peak value of 30% reported by some studies (37).

The presence of thyroid antibodies among new-onset T1D patients occurs to be a common phenomenon, which may be significant for predicting the possible clinical manifestation of AITD at the same time of setting the T1D diagnosis (37). According to a previously conducted study (38), thyroid function at T1D onset is largely affected by metabolic derangement, and the assessment of thyroid antibodies may be valuable in recognizing patients prone to developing hypothyroidism. Our findings provide further evidence that the highest number of patients diagnosed with AITD was observed among teenagers (15–18 years). We also found a solid female predominance in both thyroid autoimmunity and AITD at diagnosis of T1D. Our results are consistent with existing studies that notice thyroid autoimmunity more often in girls (33, 39) and increase by 4.6% in prevalence of hypothyroidism (including subclinical cases) for every 10-year increase of age in T1D and with diabetes duration (15). These findings correlate with overall trends of thyroid autoimmunity incidence in other countries (39, 40) and also in Poland (41). Contrary to our findings, the meta-analysis based on 14 studies shows that the risk of thyroid dysfunction is much higher in children in comparison to adults (42).

Another very interesting finding of our study is the correlation between GADA and thyroid autoimmunity. In our study, we found vital correspondence between the presence of GADA antibodies and both clinically manifested thyroid AD and asymptomatic thyroid autoimmunity. Our results are consistent with previously published data (39, 43). Glutamic acid decarboxylase antibodies are found in ~70–80% of patients who suffer from T1DM, long before the onset of clinical symptoms, and they remain positive over a long time after. The GADA presence is linked positively to gender (female) and older age at the time of diabetes onset (44). The association between GADA and TPOAb was previously observed. Glutamic acid decarboxylase in patients with T1D not only could indicate higher risk of more severe course of T1D but also could be a marker that takes part in reflecting other endocrine autoimmunity such as thyroid autoimmunity (39).

Deficiency of thyroid hormones, even subclinical, may lead to serious complications. Because of their influence on heart, vessels, and adipose tissue function, they play an important role in atherosclerotic processes (45, 46). Moreover, a possible link was found between subclinical hypothyroidism and cardiovascular risk factors and heart failure in adult patients (47). Despite the fact that similar conclusions have not been confirmed in pediatric patients yet, several studies have found some subtle proatherogenic abnormalities in children with a slight increase in TSH levels, showing improvement after levothyroxine treatment (45). All the facts mentioned above together with the results of our analyses confirm the need for screening and early treatment for AITD among T1D patients.

Celiac disease perception by clinicians has changed remarkably over past 50 years (48), and it became to be known as chronic, food-induced autoimmune disorder that is common and is diagnosed worldwide in patients of any age. Celiac disease keeps increasing in general population over last decades (49, 50). Some studies suggest that accuracy in diagnosis of CD (51) depends on the criteria used for selection of patients for the biopsy (the percentage of patients diagnosed increased with the percentage of patients qualified for biopsy) (35). Celiac disease, when untreated, could lead to malnutrition and also extraintestinal manifestations of this disease: impaired bone density and stunted growth in children, liver failure, or even infertility (52, 53). Celiac disease, except for the aforementioned complications, may lead to increased risk of occurrence of other disorders. A retrospective study reported that patients who were on a gluten-free diet for a long time developed 50% fewer number of ADs during the period up to about 15 years of follow-up (54). Because of this, screening, possibly early identification and diagnosis are undoubtedly vital. The strong association of CD and AITDs was widely described in literature (55, 56). In patients with T1D, prevalence of CD is higher (4.7%) than in general population (1.4%) (35). Similarly to these results, prevalence of CD was increased among our patients, and the growth trend over the years was also noted.

Nowadays, there is a great effort in research trying to find the possible reasons for increased and multiple autoimmunity in T1D young patients (35). One of the possible explanations could be a common genetic background and defective immunoregulation (44). As for a shared genetic background, we can observe familial aggregation of ADs. It is widely known that some haplotypes of HLA predispose to both T1D and other ADs, i.e., HLA-DQ2, DQ4, and CD (57, 58). Also both T1D and AITD represent similar susceptibility gene polymorphisms, with HLA and non-HLA variants, which may cause such a clustering (59).

The TEDDY Study Group has already assessed a number of environmental candidate triggers, which include probiotics, infections, micronutrient, and microbiome, which could be responsible for recent increase in AD incidence (60). Other possible factors are altering the balance of gut microbiota due to probiotic or antibiotics use (61, 62) and epigenetic changes induced by air pollutants (63).

The increase in incidence of ADs over the last decades is accompanied by the outbreak of obesity. Complex interplay between the metabolic and immune processes is not fully understood, but may interact in developing various disorders (64). However, there are researches describing a visible correlation between obesity and greater prevalence or a worse prognosis of numerous immune-mediated conditions. In our study, the trend in annual changes of SDS-BMI was not considered, unfortunately. Numerous researches have described the properties of white adipose tissue as a crucial site in the dissolvable mediators generation defined as “adipokines” that in majority carry a proinflammatory activity. These adipokines occur to be the link between immune system and adipose tissue (65).

## Limitations of the Study

The first and foremost limitation to our study is the retrospective design, the improvement over the years in the diagnosis of many conditions, and not testing specifically for other autoimmune conditions. Second is the fact that data were sourced from electronic medical records. Tests screening for ADs other than those mentioned in the study were not performed because of lack of specific guidelines and lack of symptoms manifested by patients accounted for in the study. Controversial finding of our study is that we noticed a lack of simultaneous significant increase in prevalence of laboratory-recognized autoimmunity. It may be caused by growth in the amount of currently unknown factors triggering the development of AD in patients with present autoimmunity. We must also admit that, over the observed years, the diagnostic methods improved, ultrasonography of the thyroid gland was introduced into routine diagnostic method in all T1D patients, and diagnostic criteria for CD changed as well. These factors may have influence on this discrepancy. Further analysis of data gathered by us is required in order to identify factors possibly responsible for the observed phenomenon.

## Conclusions

The IRR of T1D in children increased 4% a year, and the standardized IR increased 1.7-fold over the 9-year observation period. Additional autoimmunity represents a significant comorbidity in patients with new-onset T1D. The number of children diagnosed with AADs that accompany T1D is rapidly growing in all age groups throughout recent years. The most prone group of patients occurred in girls, older children, and patients who tested positive for GADA antibodies. Thus, more attention should be paid on subjects with other coexisting AD since T1D onset, and we hope that our work will contribute to greater emphasis on monitoring this problem. We believe that getting the comprehensive knowledge on this epidemiological problem with great clinical impact will help to establish better interdisciplinary treatment approach.

## Data Availability Statement

The raw data supporting the conclusions of this article will be made available by the authors, without undue reservation.

## Ethics Statement

The studies involving human participants were reviewed and approved by Medical University Bioethical Committee (no of approval: APK.002.112.2020). Written informed consent to participate in this study was provided by the participants' legal guardian/next of kin.

## Author Contributions

BG-O made substantial contributions to study design and conception, acquisition, analysis and interpretation of data, and wrote the paper. PP and KZ partially gathered the data, analyzed and interpreted it, and co-wrote the paper. MS prepared the figures, partially gathered the data, analyzed and interpreted it, and co-wrote the paper. MJ-S made substantial contributions to study conception and design, acquisition, analysis, and interpretation of data. AM performed and interpreted statistical analyses. AK, AP, and WŁ were involved in the critical revision for important intellectual content. AB was involved in the design, conception, analysis, and revised the paper. All authors contributed in discussions, read, and approved the final version of the manuscript.

## Conflict of Interest

The authors declare that the research was conducted in the absence of any commercial or financial relationships that could be construed as a potential conflict of interest.

## References

[1] Federation International Diabetes IDF Diabetes Atlas, 9th ed. Brussels (2019). Available online at: https://www.diabetesatlas.org (accessed March 2, 2020).

[2] Maahs DM West NA Lawrence JM Mayer-Davis EJ Epidemiology of type 1 diabetes. Endocrinol Metab Clin North Am. (2010) 39:481–97. 10.1016/j.ecl.2010.05.011 20723815 PMC2925303

[3] Tajima N Morimoto A. Epidemiology of childhood diabetes mellitus in Japan. Pediatr Endocrinol Rev. (2012) 10(Suppl. 1):44–50. 23330245

[4] Harjutsalo V Sund R Knip M Groop P-H. Incidence of type 1 diabetes in Finland. JAMA. (2013) 310:427. 10.1001/jama.2013.8399 23917294

[5] Tuomilehto J. The emerging global epidemic of type 1 diabetes. Curr Diab Rep. (2013) 13:795–804. 10.1007/s11892-013-0433-5 24072479

[6] Szalecki M Wysocka-Mincewicz M Ramotowska A Mazur A Lisowicz L Ben-Skowronek I. Epidemiology of type 1 diabetes in Polish children: a multicentre cohort study. Diabetes Metab Res Rev. (2018) 34:e2962. 10.1002/dmrr.2962 29144024

[7] Lerner A Jeremias P Matthias T The world incidence and prevalence of autoimmune diseases is increasing. Int J Celiac Dis. (2015) 3:151–5. 10.12691/ijcd-3-4-8

[8] Selmi C. Autoimmunity in 2010. Autoimmun Rev. (2011) 10:725–32. 10.1016/j.autrev.2011.06.004 21763468

[9] Minelli R Gaiani F Kayali S Di Mario F Fornaroli F Leandro G. Thyroid and celiac disease in pediatric age: a literature review. Acta Biomed. (2018) 89:11–6. 10.23750/abm.v89i9-S.7872 30561390 PMC6502193

[10] Hanley P Lord K Bauer AJ. Thyroid disorders in children and adolescents: a review. JAMA Pediatr. (2016) 170:1008–19. 10.1001/jamapediatrics.2016.0486 27571216

[11] Biondi B Cappola AR Cooper DS. Subclinical hypothyroidism: a review. JAMA. (2019) 322:153–60. 10.1001/jama.2019.9052 31287527

[12] van den Driessche A Eenkhoorn V Van Gaal L De Block C. Type 1 diabetes and autoimmune poly-glandular syndrome: a clinical review. Neth J Med. (2009) 67:376–87. 20009114

[13] Kahaly GJ Hansen MP. Type 1 diabetes associated autoimmunity. Autoimmun Rev. (2016) 15:644–8. 10.1016/j.autrev.2016.02.017 26903475

[14] Mahmud FH Elbarbary NS Fröhlich-Reiterer E Holl RW Kordonouri O Knip M. ISPAD clinical practice consensus guidelines 2018: ocomplications and associated conditions in children and adolescents with type 1 diabetes. Pediatr Diabetes. (2018) 19:275–86. 10.1111/pedi.12740 30066458 PMC6748835

[15] Kordonouri O. Natural course of autoimmune thyroiditis in type 1 diabetes: association with gender, age, diabetes duration, and puberty. Arch Dis Child. (2005) 90:411–4. 10.1136/adc.2004.056424 15781936 PMC1720371

[16] Klonowska B Charemska D Jabłonska J Banach A Kacka A Szynkarczuk E. Carotid artery intima-media thickness (cIMT) in young type 1 diabetic patients in relation to comorbid additional autoimmune diseases and microvascular complications. Pediatr Endocrinol Diabetes Metab. (2016) 22:92–104. 10.18544/PEDM-22.03.0057 28633159

[17] Statistics Poland (Główny Urzad Statystyczny - GUS) LOCAL DATA BANK. Available online at: https://bdl.stat.gov.pl/BDL/start (accessed March 15, 2020).

[18] OLAF Project Children's Memorial Health Institute. Available online at: http://olaf.czd.pl/index.php?option=com_content&view=category&layout=blog&id=28&Itemid=75 (accessed March 15, 2020).

[19] Mayer-Davis EJ Kahkoska AR Jefferies C Dabelea D Balde N Gong CX. ISPAD clinical practice consensus guidelines 2018: definition, epidemiology, and classification of diabetes in children and adolescents. Pediatr Diabetes. (2018) 19:7–19. 10.1111/pedi.12773 30226024 PMC7521365

[20] Patterson CC Dahlquist G Soltész G Green A. Variation and trends in incidence of childhood diabetes in Europe. Lancet. (2000) 355:873–6. 10.1016/S0140-6736(99)07125-1 10752702

[21] Peczynska J Peczynska J Jamiołkowska M Polkowska A Zasim A Łuczynski W. Epidemiology of diabetes type 1 in children aged 0–14 in Podlasie Province in years 2005–2012. Pediatr Endocrinol Diabetes Metab. (2016) 22:14–9. 10.18544/PEDM-22.01.0045 28132068

[22] Chobot A Polanska J Brandt A Deja G Glowinska-Olszewska B Pilecki O. Updated 24-year trend of type 1 diabetes incidence in children in Poland reveals a sinusoidal pattern and sustained increase. Diabet Med. (2017) 34:1252–8. 10.1111/dme.13345 28257151

[23] Husby S Koletzko S Korponay-Szabó I Kurppa K Mearin ML Ribes-Koninckx C. European society paediatric gastroenterology, hepatology and nutrition guidelines for diagnosing coeliac disease 2020. J Pediatr Gastroenterol Nutr. (2020) 70:141–156. 10.1097/MPG.0000000000002497 31568151

[24] Craig ME Jefferies C Dabelea D Balde N Seth A Donaghue KC. Definition, epidemiology, and classification of diabetes in children and adolescents. Pediatr Diabetes. (2014) 15:4–17. 10.1111/pedi.12186 25182305

[25] Patterson CC Harjutsalo V Rosenbauer J Neu A Cinek O Skrivarhaug T. Trends and cyclical variation in the incidence of childhood type 1 diabetes in 26 European centres in the 25 year period 1989–2013: a multicentre prospective registration study. Diabetologia. (2019) 62:408–17. 10.1007/s00125-018-4763-3 30483858

[26] Dabelea D Mayer-Davis EJ Saydah S Imperatore G Linder B Divers J. Prevalence of type 1 and type 2 diabetes among children and adolescents from 2001 to 2009. JAMA. (2014) 311:1778–86. 10.1001/jama.2014.3201 24794371 PMC4368900

[27] You WP Henneberg M. Type 1 diabetes prevalence increasing globally and regionally: the role of natural selection and life expectancy at birth. BMJ Open Diabetes Res Care. (2016) 4:1–7. 10.1136/bmjdrc-2015-000161 26977306 PMC4780042

[28] Zhao Z Sun C Wang C Li P Wang W Ye J. Rapidly rising incidence of childhood type 1 diabetes in Chinese population: epidemiology in Shanghai during 1997–2011. Acta Diabetol. (2014) 51:947–53. 10.1007/s00592-014-0590-2 24777734

[29] PARK Y. Why is type 1 diabetes uncommon in Asia? Ann NY Acad Sci. (2006) 1079:31–40. 10.1196/annals.1375.005 17130529

[30] Sugihara S. Genetic susceptibility of childhood type 1 diabetes mellitus in Japan. Pediatr Endocrinol Rev. (2012) 10(Suppl. 1):62–71. 23330247

[31] Mayer-Davis EJ Bell RA Dabelea D D'Agostino R Imperatore G Lawrence JM. The many faces of diabetes in American youth: type 1 and type 2 diabetes in five race and ethnic populations: the SEARCH for diabetes in youth study. Diabetes Care. (2009) 32:S99. 10.2337/dc09-S201 19246580 PMC2647691

[32] Cerqueiro Bybrant M Grahnquist L Örtqvist E Andersson C Forsander G Elding Larsson H Tissue transglutaminase autoantibodies in children with newly diagnosed type 1 diabetes are related to human leukocyte antigen but not to islet autoantibodies: a Swedish nationwide prospective population-based cohort study. Autoimmunity. (2018) 51:221–7. 10.1080/08916934.2018.1494160 30444426

[33] Hughes JW Bao YK Salam M Joshi P Kilpatrick CR Juneja K. Late-onset T1DM and older age predict risk of additional autoimmune disease. Diabetes Care. (2019) 42:32–8. 10.2337/dc18-1157 30361208 PMC6300704

[34] Garmendia Madariaga A Santos Palacios S Guillén-Grima F Galofré JC. The incidence and preva-lence of thyroid dysfunction in Europe: a meta-analysis. J Clin Endocrinol Metab. (2014) 99:923–31. 10.1210/jc.2013-2409 24423323

[35] Nederstigt C Uitbeijerse BS Janssen LGM Corssmit EPM de Koning EJP Dekkers OM. Associated auto-immune disease in type 1 diabetes patients: a systematic review and meta-analysis. Eur J Endocrinol. (2019) 180:135–44. 10.1530/EJE-18-0515 30508413

[36] Orzan A Novac C Mihu M Tirgoviste CI Balgradean M. Type 1 diabetes and thyroid autoimmunity in children. Maedica. (2016) 11:308–12. 28828047 PMC5543522

[37] Jonsdottir B Larsson C Carlsson A Forsander G Ivarsson SA Lernmark A. Thyroid and islet autoantibodies predict autoimmune thyroid disease at type 1 diabetes diagnosis. J Clin Endocrinol Metab. (2017) 102:1277–85. 10.1210/jc.2016-2335 28388722 PMC5460724

[38] Balsamo C Zucchini S Maltoni G Rollo A Martini AL Mazzanti L. Relationships between thyroid function and autoimmunity with metabolic derangement at the onset of type 1 diabetes: a cross-sectional and longitudinal study. J Endocrinol Invest. (2015) 38:701–7. 10.1007/s40618-015-0248-0 25722223

[39] Jonsdottir B Larsson C Lundgren M Ramelius A Jönsson I Larsson HE. Childhood thyroid autoim-munity and relation to islet autoantibodies in children at risk for type 1 diabetes in the diabetes prediction in skåne (DiPiS) study. Autoimmunity. (2018) 51:228–37. 10.1080/08916934.2018.1519027 30486698

[40] Al-Khawari M Shaltout A Qabazard M Al-Sane H Elkum N. Prevalence of thyroid autoantibodies in children, adolescents and young adults with type 1 diabetes in Kuwait. Med Princ Pract. (2015) 24:280–4. 10.1159/000381547 25895905 PMC5588293

[41] Piatkowska E Szalecki M. Autoimmune thyroiditis in children and adolescents with type 1 diabetes. Pediatr Endocrinol Diabetes Metab. (2011) 17:173–7. 22248776

[42] Shun CB Donaghue KC Phelan H Twigg SM Craig ME. Thyroid autoimmunity in type 1 diabetes: systematic review and meta-analysis. Diabet Med. (2014) 31:126–35. 10.1111/dme.12318 24103027

[43] Jonsdottir B Andersson C Carlsson A Delli A Forsander G Ludvigsson J. Thyroid autoimmunity in relation to islet autoantibodies and HLA-DQ genotype in newly diagnosed type 1 diabetes in children and adolescents. Diabetologia. (2013) 56:1735–42. 10.1007/s00125-013-2934-9 23666211

[44] Kakleas K Soldatou A Karachaliou F Karavanaki K. Associated autoimmune diseases in children and adolescents with type 1 diabetes mellitus (T1DM). Autoimmun Rev. (2015) 14:781–97. 10.1016/j.autrev.2015.05.002 26001590

[45] Salerno M Capalbo D Cerbone M De Luca F. Subclinical hypothyroidism in childhood-current knowledge and open issues. Nat Rev Endocrinol. (2016) 12:734–46. 10.1038/nrendo.2016.100 27364598

[46] Cappola AR Ladenson PW. Hypothyroidism and atherosclerosis. J Clin Endocrinol Metab. (2003) 88:2438–44. 10.1210/jc.2003-030398 12788839

[47] Biondi B Cooper DS. The clinical significance of subclinical thyroid dysfunction. Endocr Rev. (2008) 29:76–131. 10.1210/er.2006-0043 17991805

[48] Popp A Mäki M. Changing pattern of childhood celiac disease epidemiology: contributing factors. Front Pediatr. (2019) 7:1–16. 10.3389/fped.2019.00357 31555624 PMC6727179

[49] Lebwohl B Sanders DS Green PHR. Coeliac disease. Lancet. (2018) 391:70–81. 10.1016/S0140-6736(17)31796-8 28760445

[50] Almallouhi E King KS Patel B Wi C Juhn YJ Murray JA. Increasing incidence and altered presentation in a population-based study of pediatric celiac disease in North America. J Pediatr Gastroenterol Nutr. (2017) 65:432–7. 10.1097/MPG.0000000000001532 28151767 PMC5538895

[51] Costa Gomes R Cerqueira Maia J Fernando Arrais R André Nunes Jatobá C Auxiliadora Carvalho Rocha M Edinilma Felinto Brito M. The celiac iceberg: from the clinical spectrum to serology and histopathology in children and adolescents with type 1 diabetes mellitus and down syndrome. Scand J Gastroenterol. (2016) 51:178–85. 10.3109/00365521.2015.1079645 26339731 PMC4732421

[52] Tye-Din JA Galipeau HJ Agardh D. Celiac disease: a review of current concepts in pathogenesis, prevention, and novel therapies. Front Pediatr. (2018) 6:350. 10.3389/fped.2018.00350 30519552 PMC6258800

[53] Laurikka P Nurminen S Kivelä L Kurppa K. Extraintestinal manifestations of celiac disease: early detection for better long-term outcomes. Nutrients. (2018) 10:1015. 10.3390/nu10081015 30081502 PMC6115849

[54] Cosnes J Cellier C Viola S Colombel JF Michaud L Sarles J. Incidence of autoimmune diseases in celiac disease: protective effect of the gluten-free diet. Clin Gastroenterol Hepatol. (2008) 6:753–8. 10.1016/j.cgh.2007.12.022 18255352

[55] Kurien M Mollazadegan K Sanders DS Ludvigsson JF. Celiac disease increases risk of thyroid disease in patients with type 1 diabetes: a nationwide cohort study. Diabetes Care. (2016) 39:371–5. 10.2337/dc15-2117 26681723

[56] Kahaly G Frommer L Schuppan D. Celiac disease and glandular autoimmunity. Nutrients. (2018) 10:814. 10.3390/nu10070814 29941778 PMC6073228

[57] Bao F Yu L Babu S Wang T Hoffenberg EJ Rewers M. One third of HLA DQ2 homo-zygous patients with type 1 diabetes express celiac disease-associated transglutaminase autoantibodies. J Autoimmun. (1999) 13:143–8. 10.1006/jaut.1999.0303 10441179

[58] Ayesh BM Zaqout EK Yassin MM. HLA-DQ2 and -DQ8 haplotypes frequency and diagnostic utility in celiac disease patients of gaza strip, Palestine. Autoimmun Highlights. (2017) 8:11. 10.1007/s13317-017-0099-0 29143181 PMC5688040

[59] Anaya JM Tobon GJ Vega P Castiblanco J. Autoimmune disease aggregation in families with primary Sjögren's syndrome. J Rheumatol. (2006) 33:2227–34. 17086607

[60] Rewers M Hyöty H Lernmark Å Hagopian W She J-X Schatz D The environmental determinants of diabetes in the young (TEDDY) Study: 2018 update. Curr Diab Rep. (2018) 18:136 10.1007/s11892-018-1113-2 30353256 PMC6415767

[61] Calcinaro F Dionisi S Marinaro M Candeloro P Bonato V Marzotti S. Oral probiotic administration induces interleukin-10 production and prevents spontaneous autoimmune diabetes in the non-obese diabetic mouse. Diabetologia. (2005) 48:1565–75. 10.1007/s00125-005-1831-2 15986236

[62] Vaarala O Atkinson MA Neu J. The “perfect storm” for type 1 diabetes: the complex interplay be-tween intestinal microbiota, gut permeability, and mucosal immunity. Diabetes. (2008) 57:2555–62. 10.2337/db08-0331 18820210 PMC2551660

[63] Zhao C Xu Z Wu G Mao Y Liu L Dan Y. Autoimmunity reviews emerging role of air pollution in autoimmune diseases. Autoimmun Rev. (2019) 18:607–14. 10.1016/j.autrev.2018.12.010 30959217

[64] La Cava A. Leptin in inflammation and autoimmunity. Cytokine. (2017) 98:51–8. 10.1016/j.cyto.2016.10.011 27916613 PMC5453851

[65] Versini M Jeandel PY Rosenthal E Shoenfeld Y. Obesity in autoimmune diseases: not a passive bystander. Autoimmun Rev. (2014) 13:981–1000. 10.1016/j.autrev.2014.07.001 25092612

