# Supplementary material for: Increasing Co-occurrence of Additional Autoimmune Disorders at Diabetes Type 1 Onset Among Children and Adolescents Diagnosed in Years 2010–2018—Single-Center Study
Source: Front Endocrinol (Lausanne). 2020 Aug 6;11:476. doi: 10.3389/fendo.2020.00476 (PMC7424019; doi:10.3389/fendo.2020.00476)
Supplement: Supplementary file 1 [file Table_1.pdf]

Supplementary Table 1. Incidence rate (per 100000 persons-years) of type 1 diabetes during 2010-2018 according to age (age adjusted).

**Year**	**Age Group**	**IR**	**95% CI**
2010	0-4	8.22	(2.67 - 19.17)
	5-9	19.61	(9.79 - 35.09)
	10-14	29.26	(17.62 - 45.69)
	15-18	20.16	(10.73 - 34.47)
2011	0-4	28.11	(16.38 - 45.01)
	5-9	16.09	(7.36 - 30.54)
	10-14	35.37	(22.17 - 53.55)
	15-18	9.77	(3.58 - 21.26)
2012	0-4	23.45	(12.82 - 39.34)
	5-9	35.5	(21.68 - 54.83)
	10-14	23.37	(12.77 - 39.20)
	15-18	8.59	(2.79 - 20.04)
2013	0-4	24.32	(13.30 - 40.81)
	5-9	31.08	(18.42 - 49.12)
	10-14	25.85	(14.47 - 42.63)
	15-18	12.71	(05.11 - 26.19)
2014	0-4	21.54	(11.13 - 37.63)
	5-9	28.46	(16.58 - 45.57)
	10-14	37.08	(22.96 - 56.69)
	15-18	13.31	(5.35 - 27.42)
2015	0-4	16.57	(7.58 - 31.46)
	5-9	33.06	(20.19 - 51.06)
	10-14	28.7	(16.40 - 46.60)
	15-18	11.92	(4.37 - 25.94)
2016	0-4	14.68	(6.34 - 28.92)
	5-9	26.54	(15.17 - 43.11)
	10-14	25.17	(13.76 - 42.24)
	15-18	10.33	(3.36 - 24.12)
2017	0-4	30.61	(17.83 - 49.01)
	5-9	25.18	(14.09 - 41.53)
	10-14	39.22	(24.58 - 59.38)
	15-18	14.91	(6.00 - 30.73)
2018	0-4	21.08	(10.89 - 36.82)
	5-9	43.45	(28.12 - 64.14)
	10-14	52.01	(35.09 - 74.25)
	15-18	10.95	(3.56 - 25.55)
